# Natriuretic peptides improve the developmental competence of in vitro cultured porcine oocytes

**DOI:** 10.1186/s12958-017-0258-1

**Published:** 2017-05-30

**Authors:** Yanhao Zhang, Huarong Wang, Wei Liu, Ye Yang, Xiao Wang, Zhiyuan Zhang, Qirui Guo, Chao Wang, Guoliang Xia

**Affiliations:** State Key Laboratory of Agrobiotechnology, College of Biological Sciences, China Agricultural University, Beijing, 100193 People’s Republic of China

**Keywords:** Natriuretic peptide, Oocyte, Cytoplasmic maturation, Porcine

## Abstract

**Background:**

Natriuretic peptides (NPs), brain and C type NPs (BNP and CNP), were involved in the maintenance of porcine oocyte meiotic arrest. The present study investigated the effects of NPs on developmental competence of immature porcine oocytes with follicles of different sizes.

**Methods:**

Follicular fluid NP levels were examined by radioimmunoassay. The developmental competence of porcine oocytes was evaluated by cleavage and blastocyst developmental rates after in vitro fertilization (IVF) or parthenogenetic activation (PA) of cumulus oocyte complexes (COCs), which were recovered from follicle with different sizes. NP levels were examined and classified according to the cleavage potential after IVF with COCs released from these follicles.

**Results:**

The BNP and CNP concentrations were increased with follicular size in follicular fluid and sustained at the set ranges of 3.0 – 6.0 mm follicles compared to 6.1 – 8.0 mm follicles. The oocytes developed from 3.0 to 6.0 mm follicles demonstrated increased embryo cleavage and blastocyst ratios after IVF, with an increased follicle size (*P* < 0.05). Moreover, BNP and CNP significantly promoted the blastocyst developmental rates of 3.0 – 6.0 mm follicles, but could not improve the developmental competence of oocytes from 6.1 to 8.0 mm follicles due to low NP levels. The COCs from 3.0 to 4.0 mm follicles were pre-incubated in 100 ng/ml of BNP and CNP media for 20 h before regular in vitro maturation, which demonstrated 2 to 3 folds higher developmental competencies in both PA and IVF groups compared to respective controls (*P* < 0.01).

**Conclusions:**

The effects of BNP and CNP supplementation in the pre-maturation culture media (PMC) on porcine developmental competence from COCs in follicles of different sizes were different and improved the developmental competence of porcine oocytes from small antral follicle in vitro.

**Electronic supplementary material:**

The online version of this article (doi:10.1186/s12958-017-0258-1) contains supplementary material, which is available to authorized users.

## Summary sentence

The effects of BNP and CNP supplementation in the pre-maturation culture (PMC) media on the developmental competence of cumulus oocyte complexes (COCs) recovered from porcine follicles of different sizes were different. The developmental competence of porcine oocytes from small antral follicles was improved by BNP and CNP in vitro.

## Background

In mammals, the developmental competence of oocytes is acquired through completion of both nuclear and cytoplasmic maturation [1, 2]. Oocytes in antral follicles are maintained in meiotic arrest, known as the germinal vesicle (GV) stage, for a prolonged period of time from diplotene stage of prophase I till the preovulatory stage [2]. During the retrieval process of oocytes from their follicles, the GV-stage oocytes spontaneously undergo nuclear maturation in vitro, where it progresses through the first meiotic division and reach till the second metaphase (MII). These oocytes, however, attain nuclear maturation before reaching optimal cytoplasmic maturity [3, 4]. Previous studies have demonstrated that only a small portion of in vitro matured (IVM) oocytes develop into blastocysts, whereas those oocytes matured in vivo counterparts have much higher embryo developmental rates in several species [2, 4]. This indicated poor nuclear and/or cytoplasmic maturation in the current oocyte IVM systems [2–4].

To ensure developmental competence, both the nuclear and cytoplasmic maturation of oocytes should co-ordinate and proceed independently [2]. Cytoplasmic maturation is not as clearly defined as the meiotic process as the process of cytoplasmic maturation involves organelle reorganization, cytoskeleton dynamics and molecular maturation during oocyte growth and meiosis [5]. It is believed that an extension of the culture period by blocking meiosis progression could allow the completion of intraoocyte cytoplasmic changes and oocyte development [4, 6, 7]. The prematuration culture (PMC) of oocytes with pharmacological phosphodiesterase 3 (PDE3) specific inhibitors such as cilostamide and rolipram [4, 8] retards nuclear maturation, which in turn allows time for ooplasmic maturation to catch up and results in better synchronization of nuclear and cytoplasmic compartments [6, 7, 9]. Therefore, optimizing the PMC conditions may help to refine the culture output for clinical use.

The NPs are a family of widely distributed, but evolutionarily conserved, polypeptide mediators that exert a range of effects throughout the body [10]. Atrial natriuretic peptide (ANP) was discovered by showing that atrial extracts contain a potent blood pressure decreasing component [11]; whereas brain natriuretic peptide (BNP) was originally purified and sequenced from extracts of porcine brain tissue and hence it was named “brain natriuretic peptide” [12]; and C-type natriuretic peptide (CNP) was initially purified and sequenced from porcine brain extracts [13]. There is growing realization that NP actions go far beyond volume and blood pressure homeostasis. Their pleiotropic effects include a significant role in regulating the female reproduction [14]. Of all the three NP types, CNP is mainly synthesized inside the mural granulosa cells and its cognate receptor (NPR2) is present in both the cumulus and granulosa cells [8, 15–18]. CNP functions in blocking the meiosis in caprine, mouse and bovine [1, 8, 14], whereas in porcine, both BNP and CNP are required for porcine oocyte meiotic arrest [16, 19, 20]. The NPs are secreted into the follicular fluid and are held at appropriate concentrations until ovulation. However, whether these NPs contribute to oocyte cytoplasmic maturation has not been demonstrated in vivo till date.

Binding of CNP to NPR2 is primarily expressed in the cumulus cells and in turn increases cyclic guanosine monophosphate (cGMP) concentrations in the cumulus cells. Through the gap junctions, cGMP diffuses into the oocytes and inhibits the activity of PDE3A, thus preventing the degradation of cyclic adenosine monophosphate (cAMP). Elevated cAMP levels maintain meiotic arrest until the preovulatory luteinizing hormone (LH) surge induces the resumption of meiosis [1, 21]. Increasing the cAMP concentration during IVM of porcine oocytes improves maturation of cumulus and subsequent fertilization in vitro [22]. Natriuretic peptide precursor C (NPPC) promotes gap junction-mediated communication (GJC) between the oocyte and surrounding cumulus cells [8]. And bidirectional communication between oocytes and follicle cells ensure oocyte developmental competence [4, 23]. However, controversy still remains in the improvement of blastocyst rate of in vitro cultured oocytes in between bovine and caprine or mouse [1, 8, 21].

The present study examines the presence of NPs in the follicles of different sizes. The data from different concentrations of BNP and CNP in the porcine follicular fluid of cleaved embryos and non-cleaved embryos, which resulted from individually culturing of cumulus oocyte complexes (COC) from single follicle in vitro following parthenogenetic activation (PA) or in vitro fertilization (IVF) has been presented. The different effects of NPs on the developmental competence of immature porcine oocytes in follicles of different sizes were evaluated. The results implied that the oocyte developmental competence was more significantly improved by PMC of oocytes collected from the small antral porcine follicles with NPs at physiological concentration.

## Methods

### Chemicals

Unless specified, all chemicals were purchased from Sigma-Aldrich (St. Louis, MO, USA). The pregnant mare’s serum gonadotropin (PMSG) and human chorionic gonadotropin (hCG) were obtained from Sansheng Pharmaceutical Co. Ltd. (Ningbo, China).

### Animals, oocyte collection, classification and measurement

Ovaries were obtained from 6- to 12-month-old gilts at a local slaughterhouse, which were transported to our laboratory in phosphate buffered saline supplemented with 100 U/mL penicillin (Huabei Medical, Jizhou, China) and 50 mg/mL streptomycin sulfate (Sigma, St. Louis, MO) at 38.5 °C. Cells used in vitro were derived from these tissues.

The follicles were dissected from the ovarian cortex at room temperature using scissors, scalpels and tweezers. During dissection, follicles were kept in washing medium [TCM-199 with Hank’s salts supplemented with 10% of fetal calf serum (FCS)]; GIBCO BRL, Burlington, ON, Canada) on a warm plate at 38.5 °C. The follicles were measured using a graduated eyepiece (OSM-4; Olympus, Tokyo, Japan) and were classified morphologically and divided into groups according to their diameter: 3.0–4.0, 4.1–5.0, 5.1–6.0 and 6.1-8.0 mm. The criteria used for follicle selection were: (1) the presence of extensive and fine vascularisation; and (2) a shiny and translucent appearance. After follicular rupture, the presence of granulosa cells with a regular and healthy appearance and no free-floating particles in the follicular fluid were also used as a selection criterion. COCs were released by rupturing the follicles. Only COCs with a homogeneous granulated cytoplasm and at least three layers of compact of cumulus cells were used in the present study. For measurement, COCs were denuded by repeated pipetting and oocyte diameter was evaluated with a graduated eyepiece.

### Pre-maturation culture (PMC)

The pre-maturation culture of the oocyte was designed to retard nuclear maturation during the in vitro porcine oocyte culture. BNP-32 (catalog # B6651) and CNP-22 (catalog # N8768) were purchased from Sigma. The culture medium was divided into two portions for different assays. Briefly, the PMC medium was composed of basal medium plus BNP (PMC- BNP) or CNP (PMC- CNP) at a final concentration of 100 ng/mL or cilostamide (10 μM) [24] in the culture medium. All cultures were carried out in a humidified atmosphere at 38.5 °C with 5% CO_2_ in air. All COCs were cultured singly in 20 μL droplets covered with mineral oil.

### Porcine COCs culture and assessment of nuclear maturation

The basal culture medium containing 0.5 mL of TCM199 medium (Gibco, Life Technologies, CA, USA) was supplemented with sodium pyruvate (0.23 mmol/L; Sigma), glutamine (2 mmol/L; Sigma), lyophilized crystalline BSA (3 mg/mL; Sigma), penicillin G (100 U/mL; Huabei Medical, Jizhou, China) and streptomycin sulfate (50 mg/mL; Sigma). For FSH-induced maturation assay, the COCs were cultured in the above medium supplemented with FSH (0.05 U/mL; Sigma).

After the culture preparation, the oocytes were harvested, fixed in acetic acid/ethanol (1/3 v/v) for 48 h, and stained with 1% (w/v) orcein prior to phase contrast microscopic examination (200×) for evaluation of chromatin configuration. The oocyte nuclear maturation status was classified as: 1) GV stage oocyte when displaying a germinal vesicle; 2) germinal vesicle breakdown (GVBD) stage oocyte when displaying a germinal vesicle breakdown and condensed chromatin or metaphase II (M II) stages were identified by the presence of an extruded polar body.

### In vitro maturation (IVM) and In vitro fertilization (IVF) by standard

IVF of the porcine oocyte was carried as described previously. Briefly, the fertilization medium used was modified Pig-FM consisting of NaCl (90 mM; Sigma), KCl (12 mM; Sigma), NaHCO_3_ (25 mM; Sigma), NaH_2_PO_4_ (0.5 mM; Sigma), MgSO_4_ (0.5 mM; Sigma), sodium lactate (10 mM; Kanto Chemical Co., Inc., Tokyo, Japan), and 10 mM Hepes modified further by Suzuki [25] by the addition of CaCl_2_ (8 mM; Sigma), sodium pyruvate (2 Mm; Sigma), caffeine (2 mM; Sigma) and BSA (5 mg/mL; fraction V; Sigma). The same batch of frozen semen from the epididymis of a single boar (Beijing swine breeding center) was used for these experiments.

Frozen thawed spermatozoa were pre-incubated for 15 min at 38.5 °C in TCM199 with Earle’s salts adjusted to pH 7.8. A portion (10 μL) of the pre-incubated spermatozoa was introduced into 90 μL of Pig-FM containing 20 COCs showing expanded cumulus cells. The final sperm concentration was adjusted to 1 × 10^5^ cells/mL. The COCs were co-incubated with spermatozoa at 38.5 °C under 5% CO_2_ for 5 h. After co-incubation, the oocytes were freed from the surrounding cumulus cells and attached spermatozoa, and transferred into the porcine zygote medium (PZM-3; IFP, Yamagata, Japan) [26], covered with mineral oil, and kept under 5% CO_2_ atm at 38.5 °C. The presumptive zygotes were cultured in PZM-3 before evaluation of their qualities showing a homogeneous or saturated density of lipid droplets and intact zona pellucida. The percentages of embryos cleaved at or beyond the 2-cell stage, and those that developed into blastocysts, were assessed under a stereomicroscope 2 or 7 days after insemination, respectively. At the end of culture, an embryo with clear blastocoele was defined as a blastocyst.

### Parthenogenetic activation (PA)

PA of cultured porcine oocyte was performed according to Kwak et al. [26]. Briefly, after in vitro maturation, the MII-stage oocytes of each group were denuded as described previously and washed twice with activation media (280 mM mannitol solution containing 0.01 mM CaCl_2_ (Sigma) and 0.05 mM MgCl_2_ (Sigma). The oocytes were transferred in between the electrodes (ECM2001, BTX, USA), covered with the activation medium, and activated with two pulses of 120 V/mm direct current for 60 microseconds. Then the oocytes were cultured in PZM-3 covered with mineral oil under 5% CO_2_ at 38.5 °C for 7 days for evaluating their developmental competence.

### RNA extraction and quantitative real-time PCR

The follicles visible on the surface of the ovaries were isolated mechanically. Cumulus oocyte complexes (COCs) were punctured out from follicles. The cumulus and granulosa cells were pipetted and stored at −80 °C until RNA extraction. For each follicle size group, four or five pooled samples from 20 follicles and their corresponding cumulus cells were stored for subsequent analysis.

Total RNA was isolated using TRIzol (Life Technologies, Carlsbad, CA) according to the manufacturer’s protocol. Quantification and quality analysis of total RNA isolated from the above samples were determined using Nanodrop (Thermo Im Heiligen Feld 17, Germany). Total RNA (1 mg) from each sample was incubated for 20 min at 25 °C with 0.5 U DNase I (Invitrogen) before reverse transcription to eliminate genomic DNA contamination. RT of the RNA was carried out using oligo (dT) with Moloney Murine Leukaemia Virus Reverse Transcriptase (Promega, Madison, WI, USA) according to the manufacturer’s instructions.

Reverse transcription proceeded for 1 h at 42 °C. DNA was amplified by an initial incubation at 72 °C for 5 min followed by 24–33 cycles of denaturation at 72 °C for 30 s, annealing at different temperatures were for 30 s, which were presented in Additional file 1: Table S1, and extension at 72 °C for 30 s, and a final extension at 72 °C for 5 min. β-Actin was used as the internal standard. Amplified products were sequenced (Invitrogen, Beijing, China) to confirm specificity.

Quantitative real-time PCR (qRT-PCR) was performed in 96-well plates (Applied Biosystems, Foster City, CA) in reaction volumes of 25 μL containing 12.5 μL SYBR Green PCR Master Mix (Applied Biosystems, Foster City, CA), 15 ng cDNA, appropriate primers (Additional file 1: Table S1), and nuclease-free water. PCR was performed on an ABI 7500 Sequence Detection System (Applied Biosystem, Foster City, CA) using the following parameters: 10 min at 95 °C followed by 40 cycles of 15 s at 95 °C and 1 min at 60 °C. All test data were normalized to levels of glyceraldehyde-3-phosphate dehydrogenase (GAPDH) transcription. A melt curve was generated after PCR at temperature increments of 0.5 °C every 2 cycles (62 cycles total) starting at 65 °C, with fluorescence acquisition after each step. The PCR products were separated by electrophoresis in a 1% agarose gel and visualized by ethidium bromide staining. Relative intensities were quantified using Gel-Pro Analyzer 4.0 software (Media Cybernetics, Inc., Bethesda, MD, USA). The relative target gene expression level in each sample was calculated using the 2^-ΔΔ^Ct formula, as described previously [16].

### Radioimmunoassay (RIA) for detection of BNP-26 and CNP-22 in porcine follicular fluid

Porcine follicular fluid was centrifuged at 10,000 g at 4 °C for 15 min and the supernatants were collected in the centrifuge tubes containing aprotinin (Phoenix Biotech; catalog #RK-APRO; 0.6 TIU/ml) and stored at −80 °C prior to the assay. Natriuretic peptide levels in the follicular fluid were measured with the aid of commercial RIA kits: porcine BNP-26 (Sigma, #T-011-10) and CNP-22 (Sigma, Human, Rat, Mouse, Porcine; #T-012–03). These RIA kits showed no cross reactivity with each other. The sensitivity of the BNP assay was 47.1 pg/mL and that of the CNP assay was 17.9 pg/mL. In our experiments, BNP was measured with an intra-assay variation of less than 4.3% and an inter-assay variation of less than 8.7%. CNP was measured with an intra-assay variation of 5% and an inter-assay variation of 12.2%.

### Culture model individual COC from single follicle and single embryo culture in vitro

A single COC in vitro culture model was used for tracking the developmental fate of the oocyte (Fig. 2, a), where the culture medium drops were connected to each other by culture medium with composed net was designed. A single COC/embryo in one droplet that was surrounded by neighboring and connected droplets of every 10 COCs/embryos were placed. The bridge of culture medium between single COC/embryo-containing drops and groups of 10 COCs/embryos-containing drops was made of culture medium, which is considered to be the same as the single COC/embryo or 10 COCs/embryos-containing drops. The cleavages and blastocyst rates of oocytes cultured in this culture model were higher than a single COC that was placed in one droplet. The concentrations of BNP and CNP in the follicular fluid of cleaved embryos and non-cleaved embryos that resulted from culturing individual COCs from single follicle in vitro following PA or IVF were measured. The individual culture system allowed for investigating the correlation of developmental ability of each COC with the concentrations of BNP and CNP in the original follicle.

### IVM of Small antral follicle-derived COCs

The criteria used for selection of porcine small antral follicles (3.0 – 4.0 mm) (Fig. 4, a) were shiny and translucent in appearance. After follicular rupture, the presence of granulosa cells with a regular and healthy appearance and no free-floating particles in the follicular fluid were also used as a selection criterion. The COCs from small antral follicle were cultured either by standard IVM for 46 h or 66 h (as negative controls), or pre-cultured with PMCI–cilostamide (10 μM) 20 h plus IVM 46 h (as positive controls) and pre-cultured with PMCI–BNP/CNP (100 ng/mL) 20 h plus IVM 46 h (as experimental groups). The developmental competence of oocytes was investigated by PA and IVF assays.

### Experimental design

#### Expression of *NPPB* and *NPPC* mRNAs in the cumulus and granulosa cells as well as the NP levels inside the follicular fluid with follicular size, and could be associated with the oocyte developmental competence

In order to study the relationship between NP levels and oocyte developmental competence in follicles of 3.0 - 8.0 mm in size, the follicles were dissected and distributed into four groups according to the diameter and were as follows: 3.0 - 4.0, 4.1 - 5.0, 5.1 - 6.0 and 6.1 - 8.0 mm. IVF and IVM was performed with the help of standard COCs from follicles of different sizes to evaluate oocyte developmental competence. In the meantime, the *NPPB*, *NPPC* and *NPR2* gene expression levels in the somatic cells of the follicles were examined by using real-time polymerase chain reaction (PCR) and the NP levels inside the follicular fluids were examined by the radioimmunoassay (RIA).

#### The concentrations of BNP and CNP in the follicular fluid containing oocytes that generated cleaved embryos and non-cleaved embryos

To determine the correlation of developmental competence of each COC to the BNP and CNP concentrations in the original follicle, a single COC in vitro maturation was performed in a particular culture system as described above. To examine if single follicles have various developmental outcomes with different concentrations of NPs within the follicular fluid, the NP levels in each follicular fluid was examined and classified according to the cleavage potential after IVF with COCs coming from such follicles. A single COC in vitro culture model for tracking the developmental fate of the oocyte was shown in (Fig. 2, a). After standard IVM, the oocyte developmental competence was examined by PA and IVF assays individually.

#### The different effects of NPs on the developmental competence of immature porcine oocytes from follicles of different sizes

In order to clarify the effect of whether individual or a combination of NPs on the developmental competence, a final concentration that reached the physiological level in order to achieve similar inhibitory effects on oocyte meiosis with different combinations of BNP and CNP, including 100 ng/mL BNP alone; 70 ng/mL BNP + 30 ng/mL CNP; 50 ng/mL BNP + 50 ng/mL CNP; 30 ng/mL BNP + 70 ng/mL CNP; and 100 ng/mL CNP alone were added to the in vitro culture media. In order to evaluate the effects of BNP and CNP supplementation in PMC culture media on developmental competence of porcine oocytes from COCs, COCs from 3.0 - 8.0, 3.0 - 4.0, 4.1 - 6.0, 6.1-8.0 mm follicles were cultured in the presence of either by BNP, CNP or cilostamide in vitro. The porcine COCs were cultured either by standard IVM for 46 h (as negative controls), or pre-cultured with PMCI–cilostamide 20 h plus IVM 46 h (as positive controls) and pre-cultured with PMCI–BNP/CNP 20 h plus IVM 46 h (as experimental groups). The developmental competence of oocytes was investigated by PA and IVF assays.

### Statistical analysis

All experiments were performed at least three times, and values are shown as mean ± SEM. Data were analyzed by *t*-test or analysis of variance (ANOVA) with StatView software (SAS Institute, Inc., Cary, NC). The groups were compared using the Holm–Šidák test. A p value less than 0.05 was considered to be statistically significant.

## Results

### Expression of *NPPB* and *NPPC* mRNA in the cumulus and granulosa cells as well as the NP levels inside the follicular fluid with follicular size could be associated with the oocyte developmental competence

**Fig. 1:**
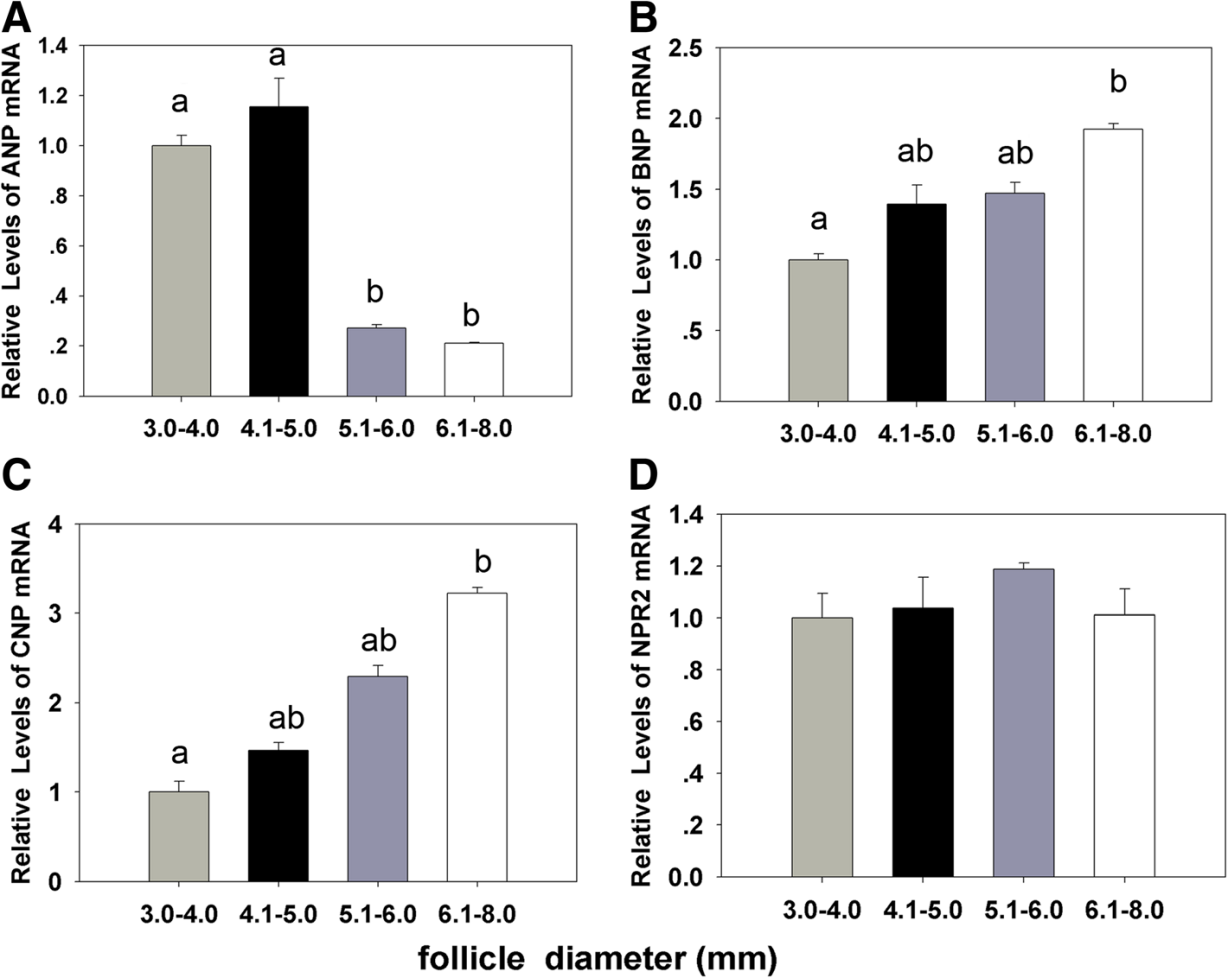
The relationship between natriuretic peptides levels and oocyte development potential in follicles sized from 3.0 to 8.0 mm. **a**, **b**, **c**, **d** The relative expressions of NPPA, *NPPB*, *NPPC* and *NPR2* genes in porcine cumulus and granulosa cells from different sized follicles. The data are the mean ± SEM and are normalized against the CYC-A gene, with results expressed relative to the control sample using the 2^-ΔΔ^ Ct method with efficiency correction. Different columns with various superscript letters differ significantly (*P* < 0.05)

The results showed that the cleavage and blastocyst rates were increased with the dimension of the follicle from 3.0 to 6.0 mm. Oocytes from follicle size of 4.1 - 6.0 mm demonstrated significant developmental competence than 6.1 - 8.0 mm in size (*P* < 0.001) (Table 1). The relative abundance of *NPPB* and *NPPC* mRNAs were increased gradually in the cumulus and granulosa cells with the follicle size (Fig. 1, b, c). But the expression of *NPPA* was decreased with the increase in the follicular diameter (Fig. 1, a). The expression of *NPR2* demonstrated no significant difference with the follicular cumulus and granulosa cells among follicles of different diameters (Fig. 1, d).

**Table 1:** The cleavage and blastocyst rates on day 2 and day 7 after IVF with the dimension of follicle from 3.0 to 8.0 mm (mean ± S. E. M.)

Follicle diameter (mm)	oocytes(n)	cleaved oocytes (%)	blastocysts (%)
3.0–4.0	215	11.2 ± 2.7^a^	3.6 ± 1.67 ^a^
4.1–5.0	201	37.5 ± 2.3 ^b^	10.1 ± 1.8 ^b^
5.1–6.0	235	41.64 ± 1.6 ^b^	13.3 ± 2.9 ^c^
6.1–8.0	206	30.9 ± 2.2 ^c^	8.3 ± 2.5^d^

^abcd^Means in the same column with different superscripts were significantly different (*p* < 0.05)

The follicles were classified according to the differences in diameter. The concentrations of both BNP and CNP in the follicular fluid were similar to each other and were sustained at set ranges (55 to 59 ng/mL for BNP and 36 to 40 ng/mL for CNP) in 3.0 - 6.0 mm follicles, but all were significantly higher (*P* < 0.05) than those of 6.1 - 8.0 mm follicles (40 ng/mL and 32 ng/mL, respectively, Table 2).

**Table 2:** The concentrations of BNP and CNP in follicular fluid from 3.0 to 8.0 mm follicle were examined by RIA assay (mean ± S. E. M.)

Follicle diameter (mm)	BNP (mg/ml)	CNP (mg/ml)
3.0–4.0	55.9 ± 2.8 ^a^	36.3 ± 2.4 ^a^
4.1–5.0	56.8 ± 5.4 ^a^	37.7 ± 3.8 ^a^
5.1–6.0	58.9 ± 3.4 ^a^	39.4 ± 2.6 ^a^
6.0–8.0	39.6 ± 6.7 ^b^	32.3 ± 2.8 ^b^

At least three follicles were examined at each time point for each experiment. ^ab^Means in the same column with different superscripts were significantly different (*p* < 0.05)

### The concentrations of BNP and CNP in the follicular fluid containing oocytes that generated cleaved embryos and non-cleaved embryos are significantly different

**Fig. 2:**
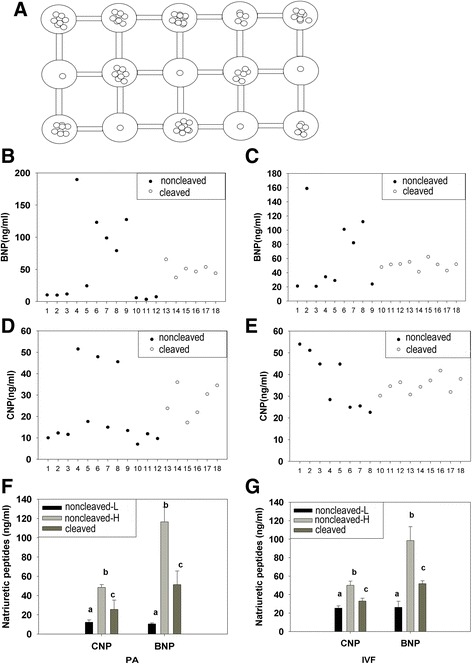
Detected natriuretic peptides concentrations in single follicles, classified according to the cleavage potential after in vitro fertilization with COCs coming from such follicles. **a** The single COCs in vitro culture model. **b**, **d** The concentrations of BNP, CNP in the follicular fluid of cleaved embryos (n = 6) and non cleaved embryos by culturing individually COCs from Single follicle in vitro and PA. **c**, **e** The concentrations of BNP, CNP in the follicular fluid of cleaved embryos (n = 9) and non cleaved embryos by culturing individually COCs from Single follicle in vitro and IVF. **f**, **g** The mean concentrations of BNP and CNP induced from **b**-**e**, in which the data from those were divided into three parts, the non-cleaved-low, the cleaved and the non-cleaved-high, as they were compared with the mean value. At least three follicles were examined at each time point for each experiment. The bars show the mean ± SEM of data from three independent experiments. Different columns with various superscript letters differ significantly (*P* < 0.05)

The concentrations of BNP or CNP in each follicle of cleaved embryos and non-cleaved embryos by culturing individual COCs from single follicle in vitro following PA or IVF individually was shown in Fig. 2, b-e. The concentrations of BNP in the follicular fluid containing oocytes that generated cleaved embryos were 30 –70 ng/mL in PA (n = 6), (Fig. 2, b) and 45–65 ng/mL in IVF (Fig. 2, c). The concentrations of CNP were 25 – 45 ng/mL (Fig. 2, d) in PA (n = 6) and 30–45 ng/mL in IVF (n = 9) (Fig. 2, e). In addition, the NP levels in non-cleaved oocytes differed obviously compared to the cleaved ones. The non-cleaved follicles were divided into two groups: a group with higher NP levels (non-cleaved-h) and a group with lower NP levels (non-cleaved-l) in comparison to the cleaved ones (Fig. 2, f, g).

### Different effects of BNP and CNP on the developmental competence of immature porcine oocytes from follicles of different size in vitro

**Fig. 3:**
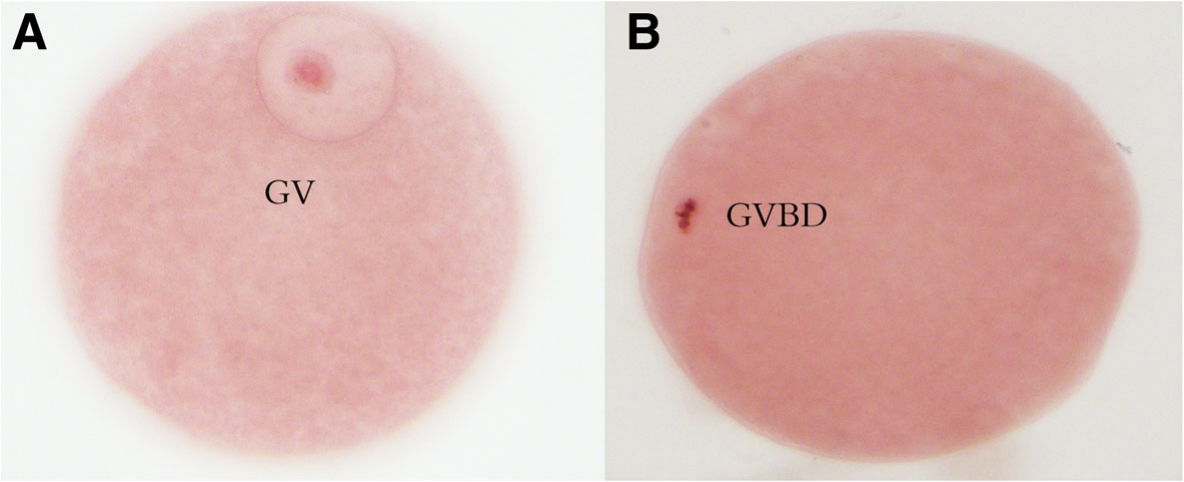
The nuclear morphological feature of porcine oocytes at different developmental stage (**a**) Oocyte at the germinal vesicle stage (GV). **b** Oocyte that has undergone germinal vesicle breakdown (GVBD)

The COCs from 3.0 to 8.0 mm follicles were used for the assay. Porcine matured oocytes were cultured in TCM199 medium with or without combining BNP and CNP with FSH for 24 h, and the effects were evaluated by examining the oocytes in the GV stage (Fig. 3, a,b). The results showed that both individual and combination of BNP and CNP have similar inhibitory effects in response to FSH stimulation of COCs (Table 3).

**Table 3:** The effects of both individual and combinations of BNP and CNP were evaluated by examining the GV stage on the resumption of FSH-induced porcine COCs meiosis (mean ± S. E. M.)

	oocytes(n)	GV(%)
Control(0 ng/mlBNP + 0 ng/mlCNP)	124	24.3 ± 1.3 ^a^
100 ng/mlBNP + 0 ng/mlCNP	143	84.3 ± 2.8 ^b^
70 ng/mlBNP + 30 ng/mlCNP	138	84.9 ± 3.2 ^b^
50 ng/mlBNP + 50 ng/mlCNP	154	83.9 ± 5.3 ^b^
30 ng/mlBNP + 70 ng/mlCNP	127	84.3 ± 3.5 ^b^
0 ng/mlBNP +100 ng/mlCNP	159	85.3 ± 2.1 ^b^

At least three independent experiments were assessed at each point for each assay. ^ab^Means in the same column with different superscripts were significantly different (*p* < 0.01)

**Fig. 4:**
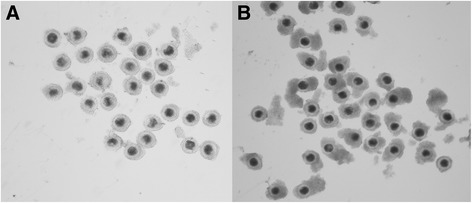
COCs recovered from (**a**) the 3.0 - 4.0 mm antral follicles, (**b**) the 4.1 - 8.0 mm antral follicles

After IVF and embryo culture, the COCs from 3.0 to 8.0 mm follicle showed no significant differences in the blastocyst rate in different treatments (Table 4). In the IVF experiment, the oocyte cleavage rates of COCs from 3.0 to 4.0 mm was significantly (*P* < 0.01) higher in the PMC–BNP/CNP group compared to the controls (Table 5). Similar to the cleavage rates, the blastocyst development rates were significantly higher in both BNP (12.6%) and CNP (11.6%) groups or cilostamide (8.8%) than the controls, including 46 h (4.2%) (*P* < 0.01) (Table 5). The phenomenon was more obvious with PA experiment (*P* < 0.01) [BNP (31.7%), CNP (32.0%), cilostamide (25.6%), control (12.6%)]. The cleavage as well as blastocyst formation rates of BNP/CNP supplement groups were also significantly higher than the controls (Table 5). Similar to the cleavage rates, the blastocyst developmental rates of oocytes recovered from the 4.0 – 6.0 mm follicles were increased significantly in the PMC–BNP/CNP group than the controls (*P* < 0.05) [BNP (16.5%),CNP (17.5%), cilostamide (15.2%), control (13.5%)] (Table 6). But the blastocyst developmental rates of oocytes were decreased significantly (*P* < 0.05), which showed BNP (5.1%), CNP (5.0%), cilostamide (4.7%), control (8.3%) from 6.0 to 8.0 mm in the PMC–BNP/CNP group than the controls (Table 7). As a result, the different effects of NPs on developmental competence in immature porcine oocytes from follicles of different sizes in vitro, especially the developmental competence of oocytes from 3.0 to 4.0 mm (Fig. 4, a) in both PA and IVF groups were 2 to 3 folds higher than the controls (*P* < 0.01). These results indicated that BNP or CNP seem to be effective for improving the developmental competence of oocytes from such follicles.

**Table 4:** The effects of NPs on the developmental competence of immature porcine oocytes from 3–8 mm follicles (mean ± S. E. M.)

	oocytes(n)	cleaved oocytes (%)	blastocysts (%)
Control	305	36.0 ± 1.9	10.2 ± 2.6
cilostamide	318	36.9 ± 2.6	11.4 ± 3.3
BNP	369	36.6 ± 3.8	9.8 ± 1.7
CNP	256	37.2 ± 1.6	11.6 ± 3.6

Cleavage rates on day 2 and Blastocyst developmental rates on day 7 after IVF of the COCs recovered from the 3.0 - 8.0 mm follicles in standard IVM for 46 h (as negative controls), or pre-cultured with PMCI–cilostamide 20 h plus IVM 46 h (as positive controls) and pre-cultured with PMCI–BNP/CNP 20 h plus IVM 46 h (as experimental groups)

**Table 5:** The effects of NPs on the developmental competence of immature porcine oocytes from 3.0-4.0 mm follicles (mean ± S. E. M.)

	IVF			PA		
	Oocytes (n)	cleaved oocytes (%)	blastocysts (%)	Oocytes (n)	cleaved oocytes (%)	blastocysts (%)
Control	326	11.3 ± 1.9 ^a^	4.2 ± 1.0 ^a^	264	31.6 ± 2.5 ^a^	12.6 ± 1.1 ^a^
cilostamide	328	38.5 ± 1.7 ^b^	8.8 ± 1.7 ^b^	256	70.4 ± 3.7 ^b^	25.6 ± 1.4^b^
BNP	314	40.5 ± 2.7 ^b^	12.6 ± 2.5 ^c^	311	81.1 ± 2.3 ^c^	31.7 ± 2.3 ^c^
CNP	289	41.4 ± 2.8 ^b^	11.6 ± 1.6 ^c^	288	78.4 ± 3.2^c^	32.0 ± 2.4 ^c^

Cleavage rates on day 2 and Blastocyst developmental rates on day 7 after IVF and PA of the COCs recovered from the 3.0 - 8.0 mm follicles in standard IVM for 46 h (as negative controls), or pre-cultured with PMCI–cilostamide 20 h plus IVM 46 h (as positive controls) and pre-cultured with PMCI–BNP/CNP 20 h plus IVM 46 h (as experimental groups). ^abc^Means in the same column with different superscripts were significantly different (*p* < 0.01)

**Table 6:** The effects of NPs on the developmental competence of immature porcine oocytes from 4.1-6.0 mm follicles (mean ± S. E. M.)

	oocytes(n)	cleaved oocytes (%)	blastocysts (%)
control	214	40.5 ± 1.42 ^a^	13.5 ± 2.9 ^a^
cilostamide	267	47.2 ± 2.04 ^ab^	15.2 ± 1.5 ^ab^
BNP	245	52.1 ± 2.01 ^b^	16.5 ± 2.6 ^b^
CNP	223	51.9 ± 1.28 ^b^	17.6 ± 2.3 ^b^

Cleavage rates on day 2 and Blastocyst developmental rates on day 7 after IVF of the COCs recovered from the 4.1 - 6.0 mm follicles in standard IVM for 46 h (as negative controls), or pre-cultured with PMCI–cilostamide 20 h plus IVM 46 h (as positive controls) and pre-cultured with PMCI–BNP/CNP 20 h plus IVM 46 h (as experimental groups). ^ab^Means in the same column with different superscripts were significantly different (*p* < 0.05)

**Table 7:** The effects of NPs on the developmental competence of immature porcine oocytes from 6.1-8.0 mm follicles (mean ± S. E. M.)

	oocytes(n)	cleaved oocytes (%)	blastocysts (%)
Control	294	30.9 ± 1.1 ^a^	8.3 ± 1.7 ^a^
cilostamide	263	23.2 ± 2.3 ^b^	4.7 ± 2.4 ^b^
BNP	301	25.1 ± 1.6^b^	5.1 ± 3.3 ^b^
CNP	268	24.6 ± 1.0 ^b^	5.0 ± 1.8 ^b^

Cleavage rates on day 2 and Blastocyst developmental rates on day 7 after IVF of the COCs recovered from the 6.1 - 8.0 mm follicles in standard IVM for 46 h (as negative controls), or pre-cultured with PMCI–cilostamide 20 h plus IVM 46 h (as positive controls) and pre-cultured with PMCI–BNP/CNP 20 h plus IVM 46 h (as experimental groups). ^ab^Means in the same column with different superscripts were significantly different (*p* < 0.05)

## Discussion

In the present study, the association of BNP and CNP on the developmental competence of porcine oocyte was studied in vivo or in vitro. Firstly, we analyzed the relative abundance of genes that were potentially involved in the oocyte competence using follicle size model. This model is well-established to identify the relationship between follicle size and oocyte developmental competence [27, 28]. The relative *NPPB* and *NPPC* mRNA expressions as well as the NP levels inside the follicular fluid in relation to follicle sizes indicated that the physiological levels of NPs at the set ranges contributed to the developmental competence of oocytes in follicles of 3.0-6.0 mm in size. There was an increase in *NPPB* and *NPPC* mRNA levels in the follicles of 6.1-8.0 mm, but a decrease in protein content. According to existed studies, the granulosa cells were accumulated in large numbers during both early and late antral growth phases although the rate of accumulation declines when the follicle reaches the preovulatory phase [29, 30]. In contrast to the follicular cells, antral fluid continues to accumulate until the time of ovulation. Indeed, during the preovulatory growth phase, follicular enlargement was due to fluid accumulation [31, 32]. Therefore, the lower concentration of NPs in the follicle size 6.0 - 8.0 mm attributed to a “dilution effect” due to the accumulation of follicular fluid rather than a reduced synthesis-accumulation of the peptides.

In order to trace the fate of individual follicles of different sizes and with various NP levels inside the follicular fluid, the COCs from 3.0 to 6.0 mm follicles were cultured individually. The results have shown that whenever the NP levels achieved a set range, the prognosis of oocytes that developed till the blastocyst stage with both PA and IVF assays demonstrated more positive follicles than those that have either higher or lower NP levels. Therefore, higher NP levels in individual follicles do not necessarily warrant a better oocyte developmental competence, rather, the NP levels sustained in the physiological range are more important for follicle development. Early reports have demonstrated that porcine BNP has high affinity to human *NPRB* and rat *NPRB* [4, 33], porcine BNP is as effective as porcine CNP to activate porcine *NPRB* [16, 34]. The relationship between BNP and CNP in oocyte meiotic arrest was not clearly understood, nevertheless in this study, 100 ng/mL of either individual or mixed BNP/CNP had similar inhibitory effect on oocyte nuclear maturation of in vitro cultured COCs. The set values of NPs were proved to be 80–100 ng/mL of total NPs with 45–65 ng/mL of BNP and 30–45 ng/mL of CNP each. In this study, the follicles with set values of NPs were proved to have a higher oocyte developmental competence than the follicles whose NP values were deviated from these set values. Therefore, higher NP levels in individual follicles do not necessarily warrant a better oocyte developmental potential, rather the NP levels sustained in the physiological range are more important for follicle development.

Oocytes from small antral follicles have a poor capacity to support embryogenesis till the blastocyst stage, but oocytes from large antral follicles have higher developmental competence [2, 35–38]. Sustaining at the set ranges of NP concentrations in the whole process of follicular growth stage is important for the oocytes to acquire developmental competence, and might require a long period of time to accumulate a greater amount of maternal mRNA and protein in cytoplasm [38]. Oocytes gradually and sequentially acquire increase in the developmental competence with advanced folliculogenesis [38]. Follicles in 6.1-8.0 mm might undergo atresia that explains both the lower protein synthesis and lower developmental competence [39, 40]. The preparation before ovulation may lower NP levels [17] and demonstrated that the NPs have function in the developmental competence. The oocyte developmental competence was influenced by other factors in 3.0 - 4.0 or 6.1 - 8.0 mm follicles, while the concentrations of NPs in follicles that may not reflect oocyte developmental competence completely. So the cleavage and blastocyst rates of oocytes in 3.0 - 4.0 mm follicles did not match the cleavage and blastocyst rates of oocytes in 4.1 - 6.0 mm follicles. But the blastocyst developmental rates of oocytes recovered from 3.0 to 4.0 mm follicles showed a significant increase in the pre-culture with PMCI–BNP/CNP. The decrease in the NPs concentrations in follicles 6.1 - 8.0 mm was minimal (1–8 ng/ml), but showed a major drop in the blastocyst rate. The NPs in PMC media could not improve the blastocyst developmental rates of the oocytes in 6.1 - 8.0 mm follicles compared to the NP concentrations in the original follicle.

The deficient IVM outcome is mostly a result of the asynchrony between nuclear and cytoplasmic maturation [4, 6]. It has been hypothesized that an in vitro prematuration period of oocytes retrieved from the antral follicles might lead to an improved oocyte cytoplasmic maturation [4, 6, 8, 9, 41]. PMC can only be achieved by blocking the inevitably spontaneous nuclear maturation that starts immediately after the oocyte that has been disconnected from the somatic compartment of the follicle, and extending the period of GV-stage arrest in PMC might alleviate this asynchrony by allowing time for completion of cytoplasmic maturity [4, 6, 42, 43]. As reported, the path to cytoplasmic competence included the buildup and storage of transcripts [44–46], synthesis and accumulation of proteins, posttranslational modifications, and ultrastructural changes. The meiotic arrest can be induced pharmacologically by manipulating the intraoocyte cAMP levels [4, 6]. Consequently, the PDE inhibitors were widely used in humans [6], mouse [6], bovine [4, 8], and porcine during the PMC process. In the present study, the cleavage and blastocyst developmental rates of oocytes recovered from the 3.0 - 6.0 mm follicles was increased significantly in pre-culture with PMCI–BNP/CNP and PMCI–cilostamide. Therefore, it is reasonable to believe that NPs, which are naturally present inside the follicular fluid could be even more beneficial than pharmacologically treated for the development of PMC systems that aimed at enhancing the outcomes of IVM protocols.

Previous study has proved that applying PDE isoenzyme inhibitor (rolipram; 100 mM)) significantly increased the blastocyst development (an increase from 34 to 51% compared to the control to the blastocysts) when bovine oocytes were fertilized at 28 h [4]. However, in this research, the PMC strategy with NPs showed no obvious contribution to the blastocyst development if porcine COCs were from the 3.0 - 8.0 mm antral follicles. The mixed oocyte population collected from 3.0 to 8.0 mm follicles demonstrated that the PMC treatment has the potential to positively affect only a subset of the total collected oocytes, while others would not benefit from this culture strategy. Also, some oocytes were derived from the early atretic follicles [47, 48], and instead were negatively affected by prolongation of culture. The cleavage and blastocyst developmental rates of oocytes recovered from the 3.0 - 6.0 mm follicles was increased significantly in the pre-culture with PMCI–BNP/CNP, while that in the 6.1 - 6.8 mm was decreased. Notably, the COCs recovered from the 3.0 - 4.0 mm follicles were cultured in the same in vitro system and presented significant effects on oocyte developmental competence, and the ratio of blastocysts was elevated by 2 to 3 folds compared to the controls. Therefore, we stated here that the effect of NPs on the developmental competence of immature porcine oocytes from follicles of different sizes was different in vitro.

## Conclusions

The effect of NPs on the developmental competence of immature porcine oocytes from follicles with different size was different in vitro. In the growing follicles, especially in 3.0 - 6.0 mm follicles, the appropriate maintenance of NP concentration is important for its developmental competence. Administration of NPs to the PMC media was specifically efficient for the antral follicles of 3.0 - 4.0 mm size to achieve better oocyte developmental competence. Our findings contribute to improve the oocyte developmental competence in porcine in vitro.

## Additional file

Additional file 1:
**Table S1.** Primers used for quantitative real-time PCR. (DOC 30 kb)

## Abbreviations

ANOVA: Analysis of variance
BNP: Brain natriuretic peptide
cAMP: Cyclic adenosine monophosphate
cGMP: Cyclic guanosine monophosphate
CNP: C-Type natriuretic peptide
COC: Cumulus oocyte complexes
FCS: Fetal calf serum
GAPDH: Glyceraldehyde-3-phosphate dehydrogenase
GJC: Gap junction mediated communication
GV: Germinal vesicle
GVBD: Germinal vesicle breakdown
hCG: Human chorionic gonadotropin
IVF: In vitro fertilization
IVM: In vitro matured
NPs: Natriuretic peptides
PA: Parthenogenetic activation
PDE3 A: Phosphodiesterase 3A
PMC: Pre-maturation culture
PMSG: Pregnant mare’s serum gonadotropin
PZM: Porcine zygote medium
qRT-PCR: Quantitative real-time polymerase chain reaction
