# Supplementary material for: Natriuretic peptides improve the developmental competence of in vitro cultured porcine oocytes
Source: Reprod Biol Endocrinol. 2017 May 30;15:41. doi: 10.1186/s12958-017-0258-1 (PMC5450137; doi:10.1186/s12958-017-0258-1)
Supplement: Additional file 1: — Table S1. Primers used for quantitative real-time PCR. (DOC 30 kb) [file 12958_2017_258_MOESM1_ESM.doc]

Supplemental Table 1. Primers used for quantitative real-time PCR

| Gene | Primers | PCR cycles | Annealing temp (°C) | Product length (bp) |
| --- | --- | --- | --- | --- |
| ANP | CTTCCTCCTCGTTCTGGTGTT ACTCTGTGCTCCAATCCTGTC | 34 | 57.8 | 97 |
| BNP | TGCTCCTGTTCTTGCACCTGTTG GCTCCTGTATCCCTGGCAGTTCT | 40 | 60 | 93 |
| CNP | AGGCAACAAGAAGGGTTTGTC ACTAACATCCCAGGCCGCT | 40 | 60 | 83 |
| NPR2 | CTACTCAGGAGCCGAGAAGCAG CGCCACAATCGCCAGAGTTGAA | 40 | 60 | 151 |
| GAPDH | AGCAATGCCTCCTGCACCACCA TGAGTCCCTCCACGATGCCGAA | 40 | 60 | 82 |
